# Analysis of Knowledge About Breastfeeding Among Health Science Students in Southern Europe

**DOI:** 10.3390/children13101354

**Published:** 2026-10-05

**Authors:** Felipe Garrido, Giacomo Cavallaro, Jose-Luis Leon, Emilio Cervera-Barba, Alfonso Lechuga-Sancho, Daniela Svedin, Telmo Bretos, Angela Casanova, Maider Belintxon, Juan-Luis González-Caballero, Eduardo Villamor, Maria Lorella Gianni, Daniela Morniroli

**Affiliations:** 1 Department of Pediatrics, Clínica Universidad de Navarra, 28027 Madrid, Spain; fgarridom@unav.es (F.G.); jleonf@unav.es (J.-L.L.); dsvedinfigo@alumni.unav.es (D.S.); acasanovaes@alumni.unav.es (A.C.); 2 Neonatal Intensive Care Unit, Fondazione IRCCS Ca’ Granda Ospedale Maggiore Policlinico, 20122 Milan, Italy; maria.gianni@unimi.it (M.L.G.); daniela.morniroli@unimi.it (D.M.); 3 Facultad de Medicina, Universidad Francisco de Vitoria, 28223 Madrid, Spain; emilio.cervera@ufv.es; 4 Department of Child and Mother Health and Radiology, Medical School, University of Cádiz, 11002 Cádiz, Spain; alfonso.lechuga@gm.uca.es; 5 Biomedical Research and Innovation Institute of Cádiz (INiBICA), Research Unit, Puerta del Mar University Hospital, 11009 Cádiz, Spain; 6 Department of Preventive Medicine and Public Health, School of Medicine, Universidad de Navarra, 31008 Pamplona, Spain; tbretosazco@alumni.unav.es; 7 Department of Community, Maternity and Pediatric Nursing, Faculty of Nursing, Universidad de Navarra, 31008 Pamplona, Spain; mbelintxon@unav.es; 8 Institute for Culture and Society, Universidad de Navarra, 31008 Pamplona, Spain; 9 Department of Statistics and Operations Research, Faculty of Medicine, University of Cadiz, 11003 Cádiz, Spain; juanluis.gonzalez@uca.es; 10 MosaKids Children’s Hospital, Maastricht University Medical Center (MUMC+), School for Oncology and Reproduction (GROW), Maastricht University, 6202 AZ Maastricht, The Netherlands; e.villamor@mumc.nl; 11 Department of Clinical Sciences and Community Health, Dipartimento di Eccellenza 2023–2027, Università degli Studi di Milano, 20122 Milan, Italy

**Keywords:** breastfeeding, undergraduate medical education, undergraduate nursing education, clinical competencies

## Abstract

**Highlights:**

**What are the main findings?**

- In a multicenter survey of 342 final-year medical, nursing, and physiotherapy students in Spain and Italy, awareness of the benefits of breastfeeding was high (93.3%). Yet knowledge of practical recommendations was modest: only 57.9% identified the WHO-recommended breastfeeding duration, 67.6% knew that breastfeeding should begin immediately after birth, and fewer than half recognized on-demand feeding.
- Knowledge of lactation physiology and human milk bioactive components was uneven (e.g., microRNA 43.5%, stem cells 44.5%, microbiota 21.1%), and awareness of international frameworks was low, with only 15.4% familiar with the WHO/UNICEF Ten Steps to Successful Breastfeeding and 48.8% with the International Code of Marketing of Breast-milk Substitutes.

**What are the implications of the main findings?**

- Only 40.6% of students had received specific breastfeeding teaching, and few felt competent in counseling parents, suggesting that current undergraduate curricula in Southern Europe do not fully prepare future health professionals to support breastfeeding.
- Standardized, competency-based breastfeeding education—covering lactation biology, practical management, and international promotion policies—should be integrated into undergraduate health sciences curricula.

**Abstract:**

**Background:** Healthcare professionals play a key role in supporting breastfeeding, yet undergraduate breastfeeding education may be insufficient. This study assessed breastfeeding knowledge among final-year health sciences students in Southern Europe. **Methods:** We conducted a multicenter cross-sectional survey among final-year medical, nursing, and physiotherapy students from universities in Spain and Italy between January and June 2025. An ad hoc questionnaire, developed by an expert panel and content-validated using a modified Delphi process, assessed knowledge of breastfeeding benefits, lactation physiology, breastfeeding management, and international promotion policies. Descriptive statistics summarized responses. **Results:** A total of 342 students participated. Only 40.6% reported specific breastfeeding-related teaching during their studies, whereas 90.1% reported having participated in a course dedicated entirely to breastfeeding outside their regular coursework. Only 57.9% correctly identified WHO recommendations on breastfeeding duration, 67.6% recognized the importance of initiating breastfeeding immediately after birth, and fewer than half correctly identified on-demand feeding. Knowledge of lactation biology was variable, particularly regarding bioactive milk components. Awareness of promotion frameworks was limited: only 15.4% were familiar with the WHO/UNICEF Ten Steps to Successful Breastfeeding, and 48.8% recognized the International Code of Marketing of Breast-milk Substitutes. Self-perceived competence in counseling families was generally low. **Conclusions:** Final-year health sciences students in Spain and Italy demonstrate good awareness of the benefits of breastfeeding but show notable deficiencies in practical recommendations, lactation biology, and international promotion strategies. Standardized competency-based breastfeeding education should be strengthened within undergraduate health sciences curricula.

## 1. Introduction

Breastfeeding is globally recognized for its health, social, and economic benefits, reducing maternal and neonatal morbidity and supporting sustainable feeding practices [1].

The World Health Organization (WHO) recommends exclusive breastfeeding for the first 6 months, with continued breastfeeding alongside complementary foods up to 2 years or beyond [2]. Nevertheless, in the WHO European Region, exclusive breastfeeding at 6 months remains low, at approximately 25%, while in the UK, fewer than 1% of infants are still receiving any breast milk at around 12 months [3,4]. Both countries show low breastfeeding rates. In Spain, exclusive breastfeeding at 6 months is about 28.5%, down from 66.5% for any breastfeeding at 3 months. In Italy, exclusive breastfeeding drops from 46.7% at 2–3 months to 30% at 4–5 months, with marked regional disparities (e.g., only ~18.1% at 4–5 months in Sicily). European surveys report that 13–39% of infants are exclusively breastfed at 6 months in both countries [5,6,7,8,9]. Adequate promotion of breastfeeding has been consistently associated with a reduction in early breastfeeding cessation. The 2018 revised UNICEF Baby-Friendly Initiative has strengthened the importance of pre-service education, recognizing that responsibility for building adequate knowledge, skills and competencies for breastfeeding protection, promotion and support begins during pre-service education [10]. By embedding these competencies in health professional curricula, pre-service education plays a crucial role in promoting the adoption of evidence-based care practices essential to the health and well-being of both mothers and infants [11]. In this context, healthcare professionals are central to improving these outcomes: pediatricians, obstetricians, and nurses play a significant role in advising mothers on breastfeeding (BF). Any general practitioner and/or medical specialist may encounter mothers breastfeeding their babies in their specialty and should therefore receive solid training [12]. Indeed, a systematic review and meta-analysis demonstrated that breastfeeding educational interventions effectively improve knowledge, attitudes, and skills among nursing, midwifery, and medical students, with clinical practicums identified as a significant moderator of both knowledge and skills [13]. Although awareness of the importance of pre-service breastfeeding education has increased substantially in recent years, important gaps remain in undergraduate curricula, particularly in Southern Europe [3,14,15,16].

In Spain, undergraduate nursing competencies include maternal and infant health but do not explicitly define breastfeeding-specific competencies [14]. Consistent with this, Cervera-Gasch et al. reported that Spanish nursing students demonstrated limited knowledge of breastfeeding, highlighting the need to strengthen breastfeeding education in undergraduate nursing curricula [14]. Similarly, in Italy, undergraduate nursing competencies encompass maternal and child health but do not explicitly address knowledge and skills related to breastfeeding [17]. Likewise, neither Spain nor Italy has national standards for undergraduate medical education that specifically define breastfeeding competencies. Consequently, breastfeeding education is generally embedded within broader maternal and child health curricula rather than being delivered as a structured, competency-based component. To address this gap, professional societies, public health organizations, and national expert groups have developed recommendations and educational standards to integrate breastfeeding competencies into undergraduate health professions education [18]. A recent scoping review by Campbell et al. concluded that, although undergraduate lactation education is broadly aligned with best-practice recommendations, substantial inconsistencies remain in curriculum content, teaching methods, and competency assessment across health disciplines. Importantly, even programs specifically designed to develop breastfeeding support skills often did not improve students’ confidence, underscoring the need for standardized, competency-based pre-service education consistent with the WHO/UNICEF Baby-Friendly Hospital Initiative recommendations [2,19].

The aim of this study was to describe the breastfeeding knowledge of final-year health sciences students from Southern Europe, using a purpose-built (ad hoc) questionnaire whose content validity was established by expert consensus. The study was not designed as a psychometric validation of a new measurement instrument.

## 2. Materials and Methods

We conducted a cross-sectional survey focused on knowledge of breastfeeding among final-year students in health sciences programs at Spanish and Italian Universities (Southern Europe).

Eligible participants were students enrolled in the final year of medical, nursing, or physiotherapy programs. Participating universities were selected by convenience from institutions with which the investigators had academic collaborations in Spain and Italy; local academic authorities authorized distribution via official student email lists.

The initial item pool was drafted from WHO/UNICEF breastfeeding recommendations, the Ten Steps to Successful Breastfeeding, the International Code of Marketing of Breast-milk Substitutes, and previously published breastfeeding knowledge surveys among health sciences students [14], and was then refined through the modified Delphi process [20,21].

Content validity was established through a process involving five experts in neonatology (GC, FG), breastfeeding (MLG, AL), statistics (JLG), and medical education (FG, AL, MLG). Three successive rounds were conducted until consensus was achieved regarding item relevance, clarity, and content coverage. Consensus was defined a priori as ≥80% agreement among the expert panel on each item’s relevance and clarity, consistent with the most commonly used threshold in health sciences Delphi studies (typically 70–80%). Questionnaire revisions were implemented after each round based on anonymized expert feedback. Three of the authors (MLG, AL, JLG) responded to a previously prepared survey (FG, GC) with Likert-scale ratings and structured feedback. The questionnaire was developed in English and then translated into Spanish and Italian [20].

The questionnaire was made available in Spanish and Italian, on SurveyMonkey (SurveyMonkey Inc., San Mateo, CA, USA). The University of Navarra Ethics Committee (Spain; Code 2025.068) approved the study for both countries, including the participant information sheet and questionnaire. The survey took approximately 15 min to complete. Respondents were informed in the cover letter that participation in the survey was voluntary. Participation was anonymous and unrelated to any academic assessment; responses could not influence students’ grades or evaluations, thereby minimizing coercion and social desirability bias. Before dissemination, the questionnaire was pilot-tested among a small group of health sciences students to assess comprehensibility and completion time. Feedback obtained was incorporated into the final version. This study received no external funding for its design, conduct, data collection, or analysis. The article processing charge (APC) was covered by a grant from the Italian Ministry of Health (Ricerca Corrente), awarded in 2026 after completion of the study. The funder had no role in the design of the study; in the collection, analysis, or interpretation of data; in the writing of the manuscript; or in the decision to publish the results.

The final questionnaire consisted of 45 items organized into three main sections (Supplementary Materials). The first section included participant information and informed consent. The second section (Table 1) collected demographic and educational data, including age, sex, country and university of study, degree program, personal experience with breastfeeding, previous breastfeeding-related education and self-perceived competence in breastfeeding counseling. The third section (Table 2) assessed breastfeeding knowledge across four predefined domains: (i) benefits of breastfeeding for infants, mothers, families and society; (ii) lactation physiology and human milk composition; (iii) practical breastfeeding management and clinical recommendations; and (iv) international breastfeeding promotion and protection strategies, including the WHO/UNICEF Ten Steps to Successful Breastfeeding and the International Code of Marketing of Breast-milk Substitutes. Most items were multiple-choice questions with a single correct answer, although some questions allowed for multiple responses.

The survey was distributed via authorized email lists in Spanish and Italian universities, with the prior approval of the relevant local academic authorities, and was available from January to June 2025. The participating universities are listed in Table 1. No reminders were sent to non-respondents, and no incentives were offered for participation. All respondents who provided informed consent were included in the analysis, and none were excluded for incomplete questionnaires. Missing data were handled using available-case analysis: each item was analyzed using only participants who answered it. Consequently, denominators may vary across items, and the relevant denominator is reported alongside each result. One item on self-perceived competence in answering breastfeeding-related examination questions was conditional on having been asked such a question; because skip logic was not enforced in the survey platform, this item is reported only among respondents who indicated they had been asked a breastfeeding-related examination question (*n* = 259).

Categorical variables were analyzed using frequencies and percentages. Statistical analysis was performed using IBM SPSS Statistics, version 29 (IBM Corp., Armonk, NY, USA).

## 3. Results

From January to June 2025, 342 answers were collected, 267 (78.1%) from Spain and 75 (21.9%) from Italy. Of the 342 respondents, 299 completed the entire questionnaire, and 43 completed only the first 18 items (demographic and educational data); no respondents were excluded. Consistent with the available-case approach described in the Methods, demographic and educational items are reported with denominators up to 342. In contrast, knowledge items answered only by full responders are reported with a denominator of 299, and the relevant denominator is specified for each result. The demographic details of the respondents are summarized in Table 1.

Most of the responding final-year undergraduate students were studying medicine (291; 85.1%), while the remainder were enrolled in nursing or physiotherapy programs. Most students (308, 90.1%) reported completing a course dedicated entirely to breastfeeding outside their regular coursework. Slightly less than half of the students (139; 40.6%) reported having taken specific theoretical or practical courses or modules on breastfeeding during their undergraduate studies. A comparable proportion of students (148; 43.3%) reported feeling “moderately competent” in answering questions about breastfeeding to mothers and fathers. One in four (27.2%) reported feeling “very” or “quite” competent. In contrast, among students who reported being asked a breastfeeding-related examination question, 230/259 (88.8%) felt competent to answer it. The remaining responses, of demographic or training-related nature, are shown in Table 1.

Most students demonstrated basic knowledge of breast milk composition, its benefits, its social impact, and its suitability as the sole source of nutrition during the first months of life. Slightly more than half of the students (173; 57.9%) correctly answered the question about the recommended duration of breastfeeding as part of the infant and young child menu. Most students (279; 93.3%) knew that breastfeeding reduces the risk of infections, immune dysregulation, neoplasms, cardiovascular risk, and sudden infant death syndrome, and that it improves the infant’s psychomotor development.

Only two-thirds of the students knew that breast milk changes its composition throughout the day and during feeding (188; 62.9%). A high percentage of students knew the concept of colostrum (87.3%), transitional milk (86.0%) and mature milk (76.3%).

The responses to questions 32–37 indicate disparate knowledge of the nutrients and components found in breast milk. A similarly disparate percentage of students knew that breast milk contains biological and cellular components, namely hormones (75.6%), microRNA (43.5%), stem cells (44.5%), leukocytes (59.9%), bacteria (21.1%), or viruses (12.4%). Only 67.6% of the students knew that it is recommended to breastfeed newborns immediately after birth. Nearly half (45.8%) responded that the newborn should be fed whenever one or both parents want it.

Only 15.4% of students were familiar with the “Ten Steps to Successful Breastfeeding” proposed by UNICEF and WHO to protect, promote and facilitate breastfeeding in maternity wards. Slightly less than half (48.8%) were aware of the WHO’s development of a Code of Marketing of Breast-milk Substitutes. Regarding maternal HIV, 163 (54.5%) correctly recognized that in industrialized settings it is not an absolute contraindication to breastfeeding, whereas 116 (38.8%) considered it an absolute contraindication, and 20 (6.7%) were unsure.

A summary of all responses to the breastfeeding knowledge questions is presented in Table 2.

## 4. Discussion

### 4.1. Main Findings

This multicenter study of final-year health sciences students from Spain and Italy found that, while their knowledge of breastfeeding’s benefits and importance was generally good, gaps were observed in knowledge of lactation physiology and management, as well as in awareness of international initiatives. These descriptive findings are consistent with the possibility that undergraduate training offers a basic understanding but may not fully prepare future healthcare professionals to provide comprehensive breastfeeding support. However, the cross-sectional design does not permit conclusions about the effect of training on knowledge.

### 4.2. Knowledge of Breastfeeding Benefits and Clinical Recommendations

Over 90% of participants recognized breastfeeding’s health benefits, including protection against infections, immune disorders, cancers, cardiovascular issues, and sudden infant death syndrome. This aligns with evidence that breastfeeding also reduces risks of asthma, dermatitis, leukemia, obesity, and offers protective effects for mothers against breast and ovarian cancer, diabetes, and cardiovascular disease [22]. A similar proportion recognized maternal benefits, such as reduced postpartum hemorrhage and reduced cancer risk. Nearly 90% viewed breastfeeding as a public health issue and recognized its benefits to society and families. These findings are consistent with the interpretation that public health messages about breastfeeding are effectively reaching undergraduates and may reflect the use of breastfeeding promotion and scientific evidence in health education [23].

However, this broad awareness contrasted with modest knowledge of specific recommendations and practical aspects of breastfeeding. Only 57.9% of students correctly identified the WHO recommendation of exclusive breastfeeding for 6 months, followed by continued breastfeeding with complementary feeding up to 2 years or more, if desired [2]. This is noteworthy, as this recommendation represents one of the most fundamental principles in infant feeding guidance. Only 67.6% knew that newborns should be breastfed immediately, and less than half identified on-demand feeding. This suggests that students understand the benefits of breastfeeding but may lack the practical knowledge that healthcare professionals convey [24]. Notably, over 30% of students either recommended or had no opinion on offering water or chamomile tea during exclusive breastfeeding, contrary to the recommendation to breastfeed exclusively without additional fluids in the first 6 months [2]. Educational strategies incorporating skills-lab teaching or clinical practicums, rather than lectures alone, are consistently associated with greater gains in breastfeeding knowledge and skills, with clinical practicums identified as a significant moderator [13].

Our results align with previous studies among healthcare students. In a multicenter Spanish study of nursing students, Cervera-Gasch et al. reported an average breastfeeding knowledge score below 5 out of 10, concluding that knowledge remains insufficient despite university training. The authors found that maternity and neonatal placements significantly improved breastfeeding knowledge. Their findings, similar to ours, suggest that breastfeeding education for health undergraduates can be enhanced within Spanish universities [14].

Comparable results were reported among UK medical students. Biggs et al. found that final-year students recognized the benefits of breastfeeding but lacked confidence in providing support and counseling, with most wanting more education [3]. Our findings show that among students who reported being asked breastfeeding-related examination questions, 88.8% (230/259) felt competent in answering them. Still, only 27.2% felt somewhat or very competent in responding to parents’ questions, and 43.3% rated themselves as moderately competent. This suggests academic teaching may provide exam knowledge but not enough confidence for real-world clinical interactions.

### 4.3. Gaps in Lactation Physiology and Breastfeeding Management

Another notable finding was the uneven understanding of lactation biology. Most students correctly identified the stages of milk, including colostrum (87.3%), transitional milk (86.0%), and mature milk (76.3%), indicating a good understanding. However, knowledge was weaker regarding specific biological aspects: only 43.5% knew that microRNAs are present in breast milk, 44.5% recognized stem cells, and 21.1% knew about milk microbiota. Fewer than half answered correctly on nutrient content during the first days. These findings suggest breastfeeding is often taught as a nutritional intervention rather than a complex biological system with immunological, endocrine, microbial, and developmental roles. Human milk contains bioactive factors, including hormones, cytokines, leukocytes, immunoglobulins, lactoferrin, stem cells, oligosaccharides, microbiota, and microRNAs, that support infant immune and gut health [25].

Furthermore, breast milk extracellular vesicles contain microRNAs that regulate gene expression, thereby controlling growth, inflammation, and T-regulatory cell activation, thereby protecting against autoimmunity and necrotizing enterocolitis [26,27,28,29]. This pattern is consistent with previous studies among medical and nursing students, in which knowledge of breastfeeding benefits was stronger than knowledge of lactation physiology, breast milk composition and practical breastfeeding management [30,31]. Consequently, future healthcare professionals should be familiar not only with the nutritional role of human milk but also with its bioactive properties [32]. The finding that nearly 40% of students incorrectly considered maternal HIV infection an absolute contraindication to breastfeeding reflects an important knowledge gap. In high-income settings, exclusive formula feeding is no longer the sole recommendation: since 2023, US perinatal guidance endorses a shared decision-making approach, as breastfeeding individuals with sustained viral suppression on antiretroviral therapy (ART) transmit HIV at rates below 1% [33,34]. Current recommendations include exclusive breastfeeding for 6 months, continuous maternal ART with regular viral load monitoring, infant antiretroviral prophylaxis, and gradual weaning, with a multidisciplinary team involved [35]. WHO guidance for relevant settings recommends breastfeeding for at least 12 months and up to 24 months or longer alongside ART [35]. Recent data from Mexico further support these observations. Velasco-Lavín et al. reported that only 35.1% of medical students demonstrated sufficient knowledge of breastfeeding. As in our cohort, knowledge of infant benefits was generally satisfactory, whereas understanding of breastfeeding physiology, breast milk composition, and practical aspects such as feeding initiation, frequency, and duration was substantially lower [36].

### 4.4. Training Exposure and Self-Perceived Competence

An additional observation concerns training exposure. Although 308 students (90.1%) reported having participated in a course entirely dedicated to breastfeeding, only 139 (40.6%) reported specific curricular courses, modules, or practical sessions on breastfeeding during their studies. This discrepancy likely reflects differing interpretations of the two items: the former probably capturing any incidental or extracurricular breastfeeding-related content, and the latter formal, timetabled curricular teaching. The two figures should therefore not be read as directly comparable. Accordingly, the 40.6% curricular-teaching figure is used as the more reliable indicator of formal breastfeeding education. This limited curricular exposure may explain the knowledge deficiencies observed in the questionnaire. Attendance at breastfeeding-specific modules varied across institutions, which likely reflects the absence of national competency standards and the reliance on local, discipline-specific curricular decisions rather than a unified framework. This aligns with Australian studies showing variability and limited standardization in breastfeeding education across medical schools [15,16]. Chuisano and Anderson’s review shows variations in teaching and evaluation methods, highlighting a lack of standardization in breastfeeding education across institutions and a limited number of high-quality studies on this topic among physicians and nurses [37]. Collectively, these findings suggest breastfeeding education relies on local efforts rather than universal standards. The implications of these findings extend beyond undergraduate education, as numerous studies have shown that healthcare professionals play a critical role in initiating and sustaining breastfeeding. Mothers often cite inconsistent advice or a lack of support as barriers [38,39]. The American Academy of Pediatrics has highlighted specific barriers in the healthcare system, including limited provider awareness, knowledge, skills, and practices, as well as insufficient attention to evidence-based maternity care practices, such as immediate skin-to-skin contact at birth [40].

### 4.5. Awareness of International Breastfeeding Policies

Particularly concerning was the low awareness of international breastfeeding initiatives. Only 15.4% of participants correctly identified the WHO/UNICEF “Ten Steps to Successful Breastfeeding”, while fewer than half recognized the purpose of the International Code of Marketing of Breast-milk Substitutes. Both initiatives are central components of the Baby-Friendly Hospital Initiative and of global breastfeeding promotion policies [19]. Most international studies focus on breastfeeding benefits and management, but our results indicate that future healthcare professionals may lack knowledge of the institutional and public health frameworks supporting breastfeeding promotion. Healthcare professionals unfamiliar with these frameworks may have difficulty understanding the institutional strategies aimed at improving breastfeeding outcomes. They may be less able to actively participate in breastfeeding promotion at both the hospital and community levels. Similar deficiencies have been reported internationally. In their scoping review, Campbell et al. found that undergraduate lactation education often lacks consistency and omits policy, advocacy, and systems-based aspects of breastfeeding promotion [23]. Mulcahy et al. confirmed in a systematic review that the lack of standardization across frameworks, content, qualifications, and assessments hampers breastfeeding education and support for mothers [41]. There is a clear need for greater integration of health policy, advocacy, and Baby-Friendly Hospital Initiative principles within undergraduate curricula.

### 4.6. Implications for Undergraduate Education

Our findings suggest that future Southern European healthcare professionals favor breastfeeding and recognize its benefits, but often lack the knowledge essential for effective counseling. Emphasizing practical training, clinical exposure, communication skills, lactation physiology, and international policies is important. Building on the evidence discussed above, these experiential components are most effective when integrated into a structured, standardized curriculum rather than delivered as isolated or optional activities [41]. Establishing clearly defined breastfeeding competencies within undergraduate curricula may represent an important step toward improving future healthcare provision.

Additionally, our results are supported globally, indicating that deficiencies in breastfeeding education are not limited to Southern Europe. In Lebanon, Moukarzel et al. found that, despite positive beliefs, interns and residents showed limited knowledge and low self-efficacy in promoting breastfeeding [42]. The authors highlighted that many trainees relied on informal rather than professional sources, raising concerns about the adequacy of formal medical education in this field. Consistent with our findings, participants valued breastfeeding but lacked confidence in their ability to provide counseling and practical support. Similarly, an Australian mixed-methods study found substantial variation across medical schools in educational content, teaching methods, and clinical exposure, with most students receiving insufficient formal education on breastfeeding [15]. Likewise, a national survey conducted in the United Kingdom identified significant gaps in medical students’ preparedness to support breastfeeding mothers, despite widespread recognition of breastfeeding’s benefits [3]. Evidence from European settings points in the same direction. In a Croatian study, Franić et al. found that medical students at the University of Split had adequate knowledge of breastfeeding, but some held negative attitudes toward breastfeeding beyond 1 year and in public settings. Only those who took the elective “Breastfeeding Medicine” course showed significantly more positive attitudes, highlighting that curricular exposure influences both knowledge and attitudes [43]. Evidence from North America supports these observations. Meek et al. conducted a nationwide analysis of U.S. physician breastfeeding education, finding ongoing gaps in knowledge, management skills, and confidence, along with widespread demand for more structured training [44]. Similarly, a study among health professional students found that knowledge and attitudes towards breastfeeding remained suboptimal despite recognizing their role in supporting breastfeeding [24]. These data reinforce that current educational models often fail to equip healthcare students with the skills to translate theory into effective clinical practice.

The deficiencies identified among undergraduate health sciences students and the opportunities to improve curricula should not make us forget the role of continuing and cross-cutting medical training regarding breastfeeding.

### 4.7. Strengths and Limitations

Several limitations should be acknowledged. First, the cross-sectional design precludes assessment of causal relationships between educational exposure and breastfeeding knowledge. Second, participation was voluntary, so selection bias cannot be excluded. Third, most respondents were medical students, limiting comparisons across disciplines. Fourth, only two Southern European countries were included, which may limit generalizability. The sample size and the convenience selection of a few universities are insufficient to be representative at the national level; the findings are more likely to reflect the education delivered at the individual participating institutions than to reflect nationwide standards in Spain and Italy. Fifth, knowledge and competence were assessed through self-report rather than objective clinical evaluation. Because the survey was self-administered online, the use of generative artificial intelligence or external resources to answer knowledge items cannot be ruled out, potentially leading to an overestimation of knowledge. For one conditional item (self-perceived competence in answering breastfeeding-related examination questions), skip logic was not enforced in the survey platform; the item is therefore reported only among respondents who indicated they had been asked such a question in an examination, using the applicable denominator. Finally, the questionnaire was purpose-built and, although its content validity was established through a modified Delphi process, its psychometric properties (e.g., internal consistency and construct validity) were not formally evaluated; the results should therefore be interpreted as a description of knowledge among respondents rather than as output from a fully validated measurement instrument. In addition, two items assessing exposure to breastfeeding education appear to have been interpreted inconsistently by respondents, indicating that formal instrument validation is needed in future work.

## 5. Conclusions

Final-year health sciences students in Spain and Italy are aware of breastfeeding’s benefits but lack practical knowledge, an understanding of lactation biology, and strategies for promoting breastfeeding. Limited exposure to formal education suggests that curricula need to include standardized, competency-based breastfeeding training. Consistent with this, the US Preventive Services Task Force recommends providing behavioral counseling interventions or referrals during pregnancy and after birth to support breastfeeding, underscoring the importance of equipping future healthcare professionals with the knowledge and skills necessary to deliver such support [45].

## Figures and Tables

**Table 1:** Sociodemographic and academic characteristics of participants.

Questions	Answers *n* (%)
Gender/Sex (*n* = 342)	Male: 77 (22.5) Female: 265 (77.5) I prefer not to answer: 0 (0)
University (*n* = 342)	Universidad de Navarra (Spain): 125 (36.5) Universidad Francisco de Vitoria (Spain): 82 (24.0) Università degli Studi di Milano Statale (Italy): 75 (21.9) Universidad de Cádiz (Spain): 35 (10.2) Other (Spain): 25 (7.3)
Can you tell us in which country you were born in? (*n* = 342)	Italy: 72 (21.1) Spain: 258 (75.4) Other: 12 (3.5)
Can you tell us which age group you belong to? (*n* = 342)	17–20 years: 0 (0) 21–25 years: 315 (92.1) 26–30 years: 20 (5.8) More than 30 years: 7 (2.0)
Could you please tell us which course you are enrolled in? (*n* = 342)	Medicine: 291 (85.1) Nursing: 43 (12.6) Physiotherapy: 8 (2.3)
Can you confirm that you are enrolled in the final year of your degree? (*n* = 342)	Yes: 331 (96.8) No: 11 (3.2)
Have you ever participated in a course entirely dedicated to breastfeeding? (*n* = 342)	Yes: 308 (90.1) No: 34 (9.9)
Are you married or living with a partner? (*n* = 342)	Yes: 16 (4.7) No: 326 (95.3)
Do you have children? (*n* = 342)	Yes: 5 (1.5) No: 337 (98.5)
If you do have children, how many children do you have? (*n* = 342)	I don’t have any children: 337 (98.5) One child: 5 (1.5) 2 children: 0 (0) 3 or more children: 0 (0)
If you have children, were at least some of them breastfed by you or your partner? (*n* = 342)	I don’t have children: 337 (98.5) Yes: 3 (0.9) No: 2 (0.6) Yes, but with formula milk top-up (breast milk and formula milk): 0 (0)
During your training, have you ever attended specific courses, or modules, or practical courses on breastfeeding? (*n* = 342)	Yes: 139 (40.6) No: 187 (54.7) I don’t remember: 16 (4.7)
During your university exams to this date, have you ever been asked any questions related to breastfeeding? (*n* = 342)	Yes: 259 (75.7) No: 59 (17.3) I don’t remember: 24 (7.0)
If so, did you feel competent answering these questions appropriately? (*n* = 259)	Yes: 230 (88.8) No: 11 (4.2) I am not sure: 18 (6.9)
To what extent do you feel competent to answer questions from mothers and fathers related to breastfeeding? (*n* = 342)	Not competent at all: 19 (5.6) Slightly competent: 82 (24.0) Moderately competent: 148 (43.3) Somewhat competent: 85 (24.9) Very competent: 8 (2.3)
Do you know if you were breastfed as a child? (*n* = 342)	Yes: 297 (86.6) No: 38 (11.1) I don’t know: 7 (2.0)

Sociodemographic, academic, and breastfeeding-related characteristics of study participants. Values are presented as *n* (%).

**Table 2:** Knowledge about breastfeeding.

Questions	Answers *n* (%)
What kind of substances does breast milk contain?	Nutrients: 4 (1.3) Functional/bioactive substances: 1 (0.3) Both nutrients and functional/bioactive substances: 292 (97.7) I don’t know: 2 (0.7)
Breast milk is an appropriate food for…	All term and premature newborns (born before 37 weeks of gestation): 282 (94.3) Only for term babies: 5 (1.7) Only for infants with an adequate growth 6 (2.0) I don’t know: 6 (2.0)
For how long does the World Health Organization recommend a baby be breastfed?	In the first six months of a child’s life, exclusively, and up to the age of one year associated with complementary feeding: 100 (33.4) In the first 4 months of life exclusively and up to 2 years of age combined complementary feeding, but not beyond 2 years of age: 15 (5.0) At least until the age of six months on an exclusive basis and on a complementary basis until the age of two years and beyond if the child and mother so wish: 173 (57.9) I don’t know: 11 (3.7)
Does breastfeeding benefit the mother or the baby?	It benefits both: 290 (97.0) It benefits both, but benefits for the mother are of little relevance to her health: 7 (2.3) It benefits neither: 0 (0) I don’t know: 2 (0.7)
The effects of breastfeeding on the child include which of the following?	Reduced risk of infections and sudden infant death, increased risk of allergies and coeliac disease: 5 (1.7) Lower risk of infections and neoplastic diseases, higher risk of sudden infant death and chronic inflammatory diseases: 2 (0.7) Reduced risk of infections, immune dysregulation diseases, neoplasms, cardiovascular risk, sudden infant death, improved psychomotor development: 279 (93.3) Lower risk of infections, immune dysregulation diseases, neoplasms, cardiovascular risk, sudden infant death, low impact on psychomotor development: 11 (3.7) None of the above: 2 (0.7)
The effects of breastfeeding on the mother include which of the following?	Lower risk of postpartum hemorrhage and higher risk of breast and ovarian cancer: 6 (2.0) Reduced breast and ovarian cancer and increased risk of postpartum hemorrhage: 13 (4.3) Reduced risk of postpartum hemorrhage and breast and ovarian cancer: 273 (91.3) None of the above: 7 (2.3)
Do you think breastfeeding should be considered a public health issue?	Yes: 268 (89.6) No: 6 (2.0) It is not clear to me: 25 (8.4)
Do you think breastfeeding has socioeconomical benefits for society?	Yes: 269 (90.0) No: 8 (2.7) It is not clear to me: 22 (7.4)
Do you think breastfeeding has socioeconomical benefits for the families?	Yes: 286 (95.7) No: 4 (1.3) It is not clear to me: 9 (3.0)
Which of the following statements regarding breastfeeding is correct?	Breast milk changes in composition during the same feeding, but not throughout the day: 7 (2.3) Breast milk changes in composition throughout the day, but not during the same feeding: 87 (29.1) Breast milk changes in composition throughout the day and during the feeding: 188 (62.9) Breast milk does not change in composition because it is always stable from a nutritional point of view: 17 (5.7)
How is breast milk defined in the first 3–5 days?	Initial: 16 (5.4) Colostrum: 261 (87.3) Transitional milk: 13 (4.3) I don’t know: 9 (3.0)
How is breast milk defined between the sixth and fifteenth day?	Transitional milk: 257 (86.0) Mature milk: 7 (2.3) Stable milk: 9 (3.0) I don’t know: 26 (8.7)
How is breast milk defined as approximately from day 15 onwards?	Finished milk: 0 (0) Mature milk: 228 (76.3) Stable milk: 41 (13.7) I don’t know: 30 (10.0)
What are the most common nutrients found in breast milk? (in decreasing order of quantity)	Carbohydrates, lipids, proteins: 129 (43.1) Proteins, lipids, carbohydrates: 60 (20.1) Lipids, proteins, carbohydrates: 93 (31.1) I don’t know: 17 (5.7)
What is the amount of protein found in breast milk in the first 3–5 days of life?	Equal to the amount of protein found from day 5 onwards: 2 (0.7) It is superior to that found from day 5 onwards, because the functional protein component prevails: 118 (39.5) It is lower than that found from the 5th day onwards, because the lipid part predominates and because it is more digestible: 103 (34.4) I don’t know: 76 (25.4)
What is the function of breast milk in the first 3–5 days of life?	It has primarily a nutritional function, and secondarily, an immune function: 126 (42.1) It has firstly, an immune function, and secondarily, a nutritional function: 157 (52.5) It only has a nutritional function: 0 (0) It only has an immune function: 1 (0.3) I don’t know: 15 (5.0)
What role do oligosaccharides play in breast milk?	Functional: 10 (3.3) Nutritional: 44 (14.7) Both: 210 (70.2) I don’t know: 35 (11.7)
Which of the following components does breast milk contain? (you may select more than one correct answer)	Hormones: 226 (75.6) Micro RNA: 130 (43.5) Stem cells: 133 (44.5) Leucocytes: 179 (59.9) Bacteria: 63 (21.1) Virus: 37 (12.4) I don’t know: 52 (17.4)
Do you know if the composition of breast milk changes at the end of the day?	It contains more lipids and melatonin: 189 (63.2) Contains less lipids to easier digestion: 63 (21.1) It contains the same amount of lipids because it is nutritionally stable: 29 (9.7) It contains more lipids, but no melatonin: 18 (6.0)
During exclusive breastfeeding, is it also advisable for the baby to also drink water or chamomile tea to avoid dehydration both in warmer months as well as in cases of insufficient milk production?	Yes: 47 (15.7) No: 203 (67.9) I am not sure: 49 (16.4)
What is the first measure one must take after childbirth to favor the start of breastfeeding?	Dry and cover the baby and place them in the crib: 0 (0) Placing the baby in the fathers’ arms: 1 (0.3) Place the baby skin-to-skin on the mother’s abdomen: 293 (98.0) Using a pacifier to promote stabilization through comforting sucking: 1 (0.3) I don’t know: 4 (1.3)
When is it advisable for a mother to breastfeed her newborn for the first time?	3 h after birth: 55 (18.4) Immediately after birth: 202 (67.6) 12 h after birth: 14 (4.7) After 3 days: 2 (0.7) I don’t know: 26 (8.7)
If the newborn is breastfed, it’s expected that the feedings will be…	Every 3 h: 137 (45.8) Every 8 h: 9 (3.0) Any time the child or mother wishes: 137 (45.8) Any time the mother wishes, but baby should not be woken up if asleep: 4 (1.3) I don’t know: 12 (4.0)
Regarding breastfeeding on demand…	In some cases, it may be inadvisable, because it does not provide the child with routine and rhythm: 29 (9.7) It stimulates milk production through regular signals to the pituitary gland, which in turn stimulates the mammary gland to produce milk through prolactin: 223 (74.6) Decreased milk production due to excess dopamine production: 5 (1.7) Can cause nipple damage due to frequent feeding: 6 (2.0) I don’t know: 36 (12.0)
During the hospital stay after childbirth…	The baby should be in the nursery most of the time so that the mother can rest: 3 (1.0) Family members should stay with the child: 0 (0) Mother and child should spend as much time together as possible: 276 (92.3) The baby should remain in the nursery between feedings: 8 (2.7) I don’t know: 12 (4.0)
UNICEF and the WHO have developed some key points for the promotion of breastfeeding. The implementation of these points leads to the recognition of a hospital as Baby-Friendly Hospital. What are these key points called?	The 100 steps: 9 (3.0) The 10 steps: 46 (15.4) The 5 pillars of breastfeeding: 114 (38.1) I don’t know: 130 (43.5)
The WHO has developed the International Code of Marketing of Breast-milk Substitutes, this code…	Promotes the distribution of free samples of infant formula by health workers: 19 (6.4) It regulates and monitors the suitability of marketing techniques for breast-milk substitutes: 146 (48.8) It applies only to developing countries: 1 (0.3) There is no code: 2 (0.7) I don’t know: 130 (43.5)
In industrialized countries, maternal HIV infection is an absolute contraindication for breastfeeding?	Yes: 116 (38.8) No: 163 (54.5) I don’t know: 20 (6.7)

Distribution of responses to breastfeeding knowledge questions among health sciences students. Values are expressed as *n* (%). Questions covered breastfeeding recommendations, milk composition, maternal and infant health benefits, breastfeeding management, and public health aspects.

## Data Availability

The data presented in this study are available on request from the corresponding author.

## Supplementary Materials

The following supporting information can be downloaded at: https://www.mdpi.com/article/10.3390/children13101354/s1. English copy of the survey related to analysis of the knowledge on breastfeeding acquired by students of the Faculty of Medicine and Surgery at the end of their studies.

## Abbreviations

The following abbreviations are used in this manuscript:

BF	Breastfeeding
UK	United Kingdom
UNICEF	United Nations Children’s Fund
USA	United States of America
WHO	World Health Organization
