# Supplementary material for: Analysis of Knowledge About Breastfeeding Among Health Science Students in Southern Europe
Source: Children (Basel). 2026 Oct 5;13(10):1354. doi: 10.3390/children13101354 (PMC13703688; doi:10.3390/children13101354)
Supplement: Supplementary file 1 [file children-13-01354-s001.zip › children-4572016-supplementary.pdf]

1. Information sheet

First of all, we thank you for your interest in participating in this study.

We invite you to take part in a study exploring the knowledge of final-year medical, nursing or physiotherapy studentsregarding breastfeeding.

This study has been approved by the Research Ethics Committee of the University of Navarra and the University of Milan (Italy). Before deciding whether you want to participate in this study, it's important that you understand why this research is of interest, what your participation will involve, and how your information will be used.

Please take the time to carefully read the information provided below.

What is the purpose of the study?
To better understand the theoretical and/or practical competencies acquired by bioscience students about breastfeeding.

How will the study be conducted?
By using a questionnaire specifically designed by researchers and validated following the Delphi methodology. This questionnaire can be completed in approximately 7 minutes.

You should know that your participation in this study is voluntary and anonymous. Once you answer our questionnaire, since your response is not identifiable, it will not be possible to delete it.

Who can participate?
University students who are currently enrolled in their final year of their bachelor's degree in nursing or physiotherapy (4th year) or medicine (6th year), who wish to complete the questionnaire, and who have accessed it through the various dissemination channels of this study.
Your participation in the study is limited to completing the questionnaire. We do not collect the IP address (internet protocol address) from where you're answering.
No financial compensation is foreseen during the study.

Funding:
This study does not have any external funding.

Personal Data:
No personal data is collected in this study. It will not be possible to identify you through any communications generated by this study.

* 1. Would you accept to answer this questionnaire voluntarily?

- ◯ Yes
- ◯ No

2. Socio-demographic questions

* 2. Can you tell us your gender?

- ◯ Male
- ◯ Female
- ◯ I prefer not to specify

3. Can you tell us which University you’re studying at?

* 4. Can you tell us in which country you were born in?

- ◯ Italy
- ◯ Spain
- ◯ Ireland
- ◯ Other (Specify)

* 5. Can you tell us which age group you belong to?

- ◯ 17-20 years
- ◯ 21-25 years
- ◯ 26-30 years
- ◯ More than 30 years

* 6. Could you please tell us which course you are enrolled in?

- ◯ Medicine
- ◯ Nursery
- ◯ Physiotherapy

* 7. Can you confirm that you are enrolled in the final year of your degree? (even if they have outstanding subjects from previous years)

- ◯ Yes
- ◯ No

* 8. Have you ever participated in a course entirely dedicated to breastfeeding?

- ◯ Yes
- ◯ No

* 9. Are you married or living with a partner?

- ◯ Yes
- ◯ No

* 10. Do you have children?

- ◯ Yes
- ◯ No

* 11. How many children do you have?

- ◯ I don’t have any children
- ◯ One child
- ◯ 2 children
- ◯ 3 or more children

* 12. Were any of your children breastfed by you or your partner?

- ◯ I don’t have children
- ◯ Yes
- ◯ No
- ◯ Yes, but with formula milk top-up (breast milk and formula milk)

* 13. During your training, have you ever attended specific courses, or modules, or practical courses on breastfeeding?

- ◯ Yes
- ◯ No
- ◯ I don't remember

* 14. During your your university exams to this date, have you ever been asked any questions related to breastfeeding?

- ◯ Yes
- ◯ No
- ◯ I don't remember

* 15. If so, did you feel competent answering these questions appropriately?

- ◯ Yes
- ◯ No
- ◯ I don't remember

* 16. To what extent do you feel competent to answer questions from mothers and fathers related with breastfeeding?

- ◯ 1 = Not competent at all
- ◯ 2 = Slightly competent
- ◯ 3 = Moderately competent
- ◯ 4 = Somewhat competent
- ◯ 5 = Very competent

* 17. Do you know if you were breastfed as a child?

- ◯ Yes
- ◯ No
- ◯ I don't know

3. Assessment of breastfeeding knowledge

* 18. What kind of substances does breast milk contain?

- Nutrients
- Functional/bioactive substances
- Both nutrients and functional/bioactive substances
- I don't know

* 19. Breast milk is an appropriate food for...

- All term and premature newborns (born before 37 weeks of gestation)
- Only for term babies
- Only for infants with an adequate growth
- I don't know

* 20. For how long does the World Health Organization and UNICEF recommend that a baby be breastfed?

- In the first six months of a child's life, exclusively, and up to the age of one year associated with complementary feeding
- In the first 4 months of life exclusively and up to 2 years of age combined complementary feeding, but not beyond 2 years of age
- At least until the age of six months on an exclusive basis and on a complementary basis until the age of two years and beyond if the child and mother so wish
- I don't know

* 21. Does breastfeeding benefit the mother or the baby?

- It benefits both
- It benefits both, but benefits for the mother are of little relevance to her health
- It beniftis neither
- I don’t know

* 22. The effects of breastfeeding on the child include which of the following:

- Reduced risk of infections and sudden infant death , increased risk of allergies and coeliac disease
- Lower risk of infections and neoplastic diseases, higher risk of sudden infant death and chronic inflammatory diseases
- Reduced risk of infections, immune dysregulation diseases, neoplasms, cardiovascular risk, sudden infant death , improved psychomotor development
- Lower risk of infections, immune dysregulation diseases, neoplasms, cardiovascular risk, sudden infant death , low impact on psychomotor development
- None of the above

* 23. The effects of breastfeeding on the mother include which of the following:

- Lower risk of postpartum haemorrhage and higher risk of breast and ovarian cancer
- Reduced breast and ovarian cancer and increased risk of postpartum hemorrhage
- Redured risk of postpartum haemorrhage and breast and ovarian cancer
- None of the above

* 24. Do you think breastfeeding should be considered a public health issue?

- Yes
- No
- It is not clear to me

* 25. Do you think breastfeeding has socioeconomical benefits for society?

- Yes
- No
- It is not clear to me

* 26. Do you think breastfeeding has socioeconomical benefits for the families?

- Yes
- No
- It is not clear to me

* 27. Which of the following statements regarding breastfeeding is correct?

- Breast milk changes in composition during the same feeding, but not throughout the day
- Breast milk changes in composition throughout the day, but not during the same feeding
- Breast milk changes in composition throughout the day and during the feeding
- Breast milk does not change in composition because it is always stable from a nutritional point of view

* 28. How is breast milk defined in the first 3-5 days?

- Initial
- Colostrum
- Transitional milk
- I don’t know

* 29. How is breast milk defined between the sixth and fifteenth day?

- Transitional milk
- Mature milk
- Stable milk
- I don’t know

* 30. How is breast milk defined as approximately from day 15 onwards?

- Finished milk
- Mature milk
- Stable milk
- I don't know

* 31. What are the most common nutrients found in breast milk? (in decreasing order of quantity)

- Carbohydrates, lipids, proteins
- Proteins, lipids, carbohydrates
- Lipids, proteins, carbohydrates
- I don't know

* 32. What is the amount of protein found in breast milk in the first 3-5 days of life?

- Equal to the amount of protein found from day 5 onwards
- It is superior to that found from day 5 onwards, because the functional protein component prevails
- It is lower than that found from the 5th day onwards, because the lipid part predominates and because it is more digestible.
- I don't know

* 33. What is the function of breast milk in the first 3-5 days of life?

- It has primarily a nutritional function, and secondarily, an immune function
- It has firstly, an immune function, and secondarily, a nutritional function
- It only has a nutritional function
- It only has an immune function
- I don't know

* 34. What role do oligosaccharides play in breast milk?

- Functional
- Nutritional
- Both functional and nutritional
- I don't know

* 35. Which of the following components does breast milk contain? (you may select more than one correct answer)

- [ ] Hormones
- [ ] Micro RNA
- [ ] Stem cells
- [ ] Leucocytes
- [ ] Bacteria
- [ ] Virus
- [ ] I don't know

* 36. Do you know if the composition of breast milk changes at the end of the day...?

- It contains more lipids and melatonin
- Contains less lipids to easier digestion
- It contains the same amount of lipids because it is nutritionally stable
- It contains more lipids, but no melatonin

* 37. During exclusive breastfeeding, is it also advisable for the baby to also drink water or chamomile tea to avoid dehydration both in warmer months as well as in cases of insufficient milk production?

- Yes
- No
- I do not know

* 38. What is the first measure one must take after childbirth to favour the start of breastfeeding?

- Dry and cover the baby and place them in the crib
- Placing the baby in the fathers arms
- Place the baby skin-to-skin on the mother's abdomen
- Using a pacifier to promote stabilization through comforting sucking
- I don't know

* 39. When is it advisable for a mother to breastfeed her newborn for the first time?

- 3 hours after birth
- Immediately after birth
- 12 hours after birth
- After 3 days
- I don't know

* 40. If the newborn is breastfed, it’s expected that the feedings will be…

- Every 3 hours
- Every 8 hours
- Any time the child or mother wishes
- Any time the mother wishes, but baby should not be woken up if asleep
- I don’t know

* 41. Regarding breastfeeding on demand:

- In some cases it may be inadvisable, because it does not provide the child with routine and rhythm
- It stimulates milk production through regular signals to the pituitary gland, which in turn stimulates the mammary gland to produce milk through prolactin
- Decreased milk production due to excess dopamine production
- Can cause teat damage due to frequent feeding
- I don't know

* 42. During the hospital stay after childbirth

- ◯ The baby should be in the nursery most of the time so that the mother can rest
- ◯ Family members should stay with the child
- ◯ Mother and child should spend as much time together as possible.
- ◯ The baby should remain in the nursery between feedings
- ◯ I don't know

* 43. UNICEF and WHO have developed some key points for the promotion of breastfeeding. The implementation of these points leads to the recognition of a hospital as Baby-Friendly Hospital: What are these key points called?

- ◯ The 100 steps
- ◯ The 10 steps
- ◯ The 5 pillars of breastfeeding
- ◯ I don't know

* 44. The WHO has developed the International Code of Marketing of Breast-milk Substitutes, this code:

- ◯ Promotes the distribution of free samples of infant formula by health workers.
- ◯ It regulates and monitors the suitability of marketing techniques for breast-milk substitutes
- ◯ It applies only to developing countries
- ◯ There is no code
- ◯ I don't know

* 45. In industrialized countries, maternal HIV infection is an absolute contraindication for breastfeeding

- ◯ Yes
- ◯ No
- ◯ I don't know
